# Expression profile analysis of lncRNA and mRNA in uterosacral ligaments of women with pelvic organ prolapse by RNA-seq

**DOI:** 10.1097/MD.0000000000033429

**Published:** 2022-04-07

**Authors:** Xinrui Zhao, Ping Li, Lu Wang, Ping Zhang, Peishu Liu

**Affiliations:** a Department of Obstetrics and Gynecology, The Second Hospital, Cheeloo College of Medicine, Shandong University, Jinan, Shandong, China; b Department of Obstetrics and Gynecology, Shandong Second Provincial General Hospital, Jinan, Shandong, China; c Department of Obstetrics and Gynecology, Qilu Hospital, Cheeloo College of Medicine, Shandong University, Jinan, Shandong, China.

**Keywords:** expression profiles, lncRNA, pelvic organ prolapse, RNA sequencing

## Abstract

Pelvic organ prolapse (POP) is a conventional gynecological condition and the mechanism is not entirely clear. Although an increasing number of studies revealed that long non-coding RNAs (lncRNAs) have essential functions in many diseases, little knowledge has been acquired in POP. The current study aimed to investigate the regulatory mechanism of lncRNA in POP. In this report, we investigated the expression profile of lncRNAs and mRNAs between POP and the control groups in human uterosacral ligament (hUSL) tissues through RNA-seq. Cytoscape was used to construct a POP-specific lncRNA-mRNA network and select key molecules. This RNA-Seq analysis uncovered a total of 289 lncRNAs, and 41 lncRNAs and 808 mRNAs were differentially expressed between the POP and non-POP groups. Four lncRNAs were identified and validated by real-time PCR. The result of gene ontology (GO) and Kyoto encyclopedia of genes and genomes (KEGG) indicated that differentially expressed lncRNAs were abundant in the biological processes and signaling pathways concerned in POP. The differentially expressed lncRNAs were mainly enriched in protein binding, the single-organism cellular process, and cytoplasmic part. The network was constructed based on the correlation analyses of the abnormally expressed lncRNAs and their target proteins to imitate their interactions. Taken together, this study was the first to demonstrate the differential expression profiles of lncRNA in POP and normal tissues by sequencing technology. Our study indicated that lncRNAs could correlate with the development of POP and may be as significant genes in the diagnosis and treatment of POP.

## 1. Introduction

Pelvic organ prolapse (POP) is a common and extraordinary condition characterized by the descent of pelvic organs, including uterine prolapse, cystocele, and/or rectocele.^[1]^ The main clinical symptoms involve a series of illnesses the negatively impact the quality of life on a women psychological health and physical health.^[2]^ 40% of women between 45 and 85 show clinical evidence of POP and there will be more than 9 million patients with POP in the United States in 2050. Yearly government spends almost $1.4 billion on POP surgery between 1996 and 2005.^[3,4]^ Although numerous risk factors, including vaginal delivery, aging, estrogen deficiency, chronic cough, and obesity, have been considered the high risk of POP,^[5]^ the precise etiology and progression of POP are still unknown. Other than these environmental risk factors, it is necessary to identify new genes to elucidate the mechanism and develop novel methods to facilitate the clinical treatment of POP.

Non-coding RNA include microRNAs (miRNAs) and long noncoding-RNAs (LncRNAs), whose function is still being investigated. MiRNA expression profiling of anterior vaginal wall tissue in postmenopausal women with pelvic floor organ prolapse has been performed. The differentially expressed miRNAs were screened and the target genes were predicted, providing new insights into the development of pelvic floor dysfunctional diseases.^[6]^ LncRNAs are defined as RNA molecules with over 200 nucleotides in length but lack protein coding capacity.^[7]^ For the past few years, many researchers have shown that many lncRNAs may participate in multiple biological and physiological processes, such as cell proliferation, apoptosis, autophagy, immune response, and cancer metastasis, by binding to DNA, transcription factors, and miRNAs.^[8–10]^ LncRNA is widely involved in various pathological processes of collagen metabolism and aging degeneration. LncRNA H19 may inhibit apoptosis and promote the proliferation of hypertrophic scar fibroblasts through the miR-194/IGF1R/p38 MAPK signaling axis, thereby contributing to the progression of hypertrophic scars.^[11]^ Changes in RP11-296A18.3 expression in the degenerative disc eventually cause excessive apoptosis of cells in the degenerative pulp nucleus and are expected to be a new breakthrough in the treatment of disc degeneration.^[12]^ However, the role of lncRNAs in POP is still largely unknown at present.

**Figure 1.**
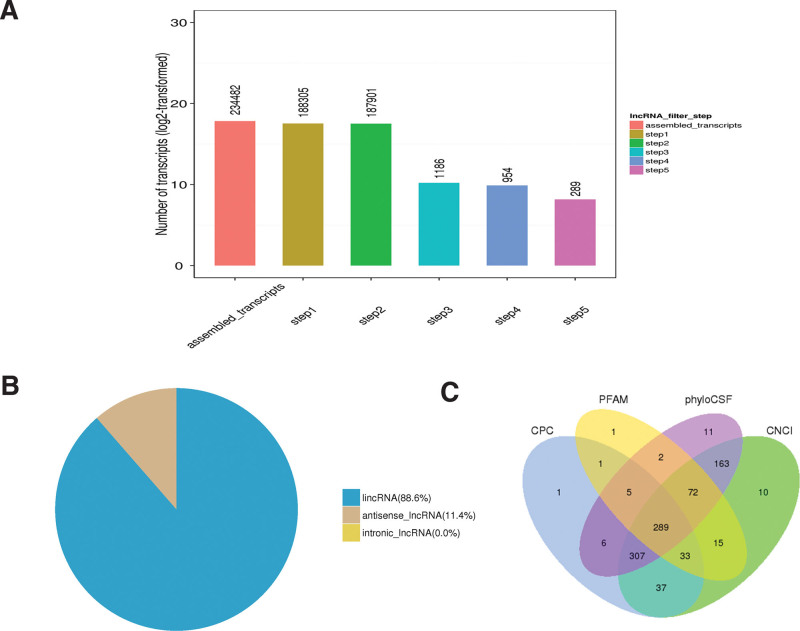
Identification of novel lncRNAs in uterosacral ligament tissues from the patients with POP and non-POP. (A) Screen of lncRNAs in human uterosacral ligament tissues. (B) Pie chart showed the classification of lncRNAs based on antisense and intronic forms. (C) Venn diagram showed the number of overlap lncRNAs. lncRNA = long noncoding RNA, POP = pelvic organ prolapse.

With the development of second generation sequencing technologies, disease excavation relies on its high-throughput information. The uterosacral ligaments are retroperitoneal structures that extend posteriorly from the uterine cervix to sacrum, defining the lateral boundaries of the rectouterine and rectovaginal spaces (Fig. 1). From an anatomical perspective, the USL can be divided into 3 parts: cervical, intermediate, and sacral sections.^[13]^ In this study, we used RNA sequencing (RNA-seq) to identify lncRNA and mRNA different expression profiles in uterosacral ligament tissues from patients with POP and non-POP. The results were confirmed via quantitative real-time polymerase chain reaction (qRT-PCR) and applied a series of bioinformatics analyses to discover the potential biological processes and signaling pathways in the pathogenesis of POP.

## 2. Methods

### 2.1. Specimen gathering and information management

The sum of uterosacral ligament specimens we adopted for our research is ten, containing 5 POP persons (POP group) and 5 non-POP controls (Control group). Women between the ages of 61.93 ± 6.63 (range, 45.00–65.00) who underwent hysterectomy for POP and other benign conditions were enrolled. The POP group underwent hysterectomy as part of pelvic reconstruction surgery for stage III, or IV POP patients according to the Pelvic Organ Prolapse Quantitation System.^[14]^ Participants with uterine prolapse and vaginal vault prolapse due to level 1 defect with or without other levels defect were included in the study under the diagnosis of the same experienced expert. The control group underwent a hysterectomy for benign diseases. Furthermore, the patients did not have any complications, including prior pelvic reconstruction surgery, chronic pelvic inflammation, cancer, cardiovascular diseases, diabetes, and immunological disorders. Women who received hormone replacement therapy were not included in the present research. Full thickness (1 cm^2^) uterosacral ligaments were harvested from 1 cm from the uterus. Pre- and post-menopause POP women were enrolled. Five pairs of POP human uterosacral ligament (hUSL) and non-POP hUSL control samples were randomly chosen for RNA-seq, and rest specimens were performed for the qRT-PCR validation experiment. The collected tissue specimens were snap-frozen in liquid nitrogen immediately and stored at −80°C until RNA isolation. All participants in the research signed the consent form. The research was endorsed by the ethics committee of Qilu Hospital of Shandong University (Approval No.: KYLL-2018 (KS)-220).

Inclusion criteria: patients with hysterectomy due to POP or other benign diseases; patients who have not used hormone drugs in recent 3 months; patients or their guardians must sign the informed consent before entering the test.

Exclusion criteria: patients undergoing hysterectomy for malignant tumor; patients who have used estrogen drugs in recent 3 months; patients with other diseases affecting collagen metabolism; patients with endometriosis and adenomyosis.

### 2.2. Total RNA isolation

Total RNA was isolated from 100 mg uterosacral ligament specimen using the TRIzol reagent (Life Technologies, Carlsbad, CA) following the supplier protocol. RNA concentration and integrity was measured by the Qubit RNA Assay Kit in Qubit 2.0 Flurometer (Life Technologies, CA) and the RNA Nano 6000 Assay Kit of the Bioanalyzer 2100 system (Agilent Technologies, CA). RNA purity was checked using the NanoPhotometer spectrophotometer (IMPLEN, CA). RNA quality was visualized on 1% agarose gels by checking bands.

### 2.3. Library construction and sequencing of lncRNA

A total of 3 μg of RNA each specimen was used for the library construction. Individual RNA-seq libraries were created for ten samples each from the POP and the control groups. After rRNA was removed by Epicentre Ribo-zero rRNA Removal Kit (Epicentre), libraries for RNA-seq were prepared using the kit following instructions. Sequencing and data collection were conducted by Novogene Bioinformatics Technology Co., Ltd. (Beijing, China).

### 2.4. Data processing

Clean data were obtained by removing reads containing adapter, reads on containing ploy-N and low quality reads from raw data. Q20, Q30, and GC content of the clean data were calculated. The mapped reads of each sample were assembled by StringTie (v1.3.1)^[15]^ in a reference-based approach.

The conditions of a novel lncRNA were as follows: the number of exon ≥ 2, length > 200 bp, FPKM ≥ 0.5 and to eliminate overlapping and coding potential transcription. Coding ability was predicted using coding-non-coding-index, coding potential calculator, Pfam-sca and phylogenetic codon substitution frequency.

### 2.5. Differential expression analysis and target gene prediction

Cuffdiff (v2.1.1) provides statistical routines for determining differential expression in digital transcript or gene expression data using a model based on the negative binomial distribution.^[16]^ In order to determine whether an lncRNA was up- or downregulated, fold change > 2 and *P* value < .05 were used.

To explore the functions of lncRNAs, we predicted their target genes through cis and trans role. Cis role was defined as the action on neighboring target genes. We searched coding genes 100k upstream and downstream of lncRNA for functional analysis. Trans role was lncRNA to identify genes based on their co-expression.

### 2.6. Gene ontology (GO) annotation and Kyoto encyclopedia of genes and genomes (KEGG) enrichment

GO annotation and KEGG enrichment analysis were used to research the effect of lncRNA targeted and differentially expressed mRNAs. Briefly, GO analysis were operated to recognize functional annotation based on the lncRNA of related cis and trans forms by the GO seq R package.^[17]^ KEGG was performed to analyze the significant pathways of cis- or trans-target genes of the lncRNAs. (http://www.genome.jp/kegg/). KEGG pathway analyses were determined using KOBAS software.^[18]^ *P* < .05 being considered as significantly enriched.

### 2.7. qRT-PCR

The selected lncRNAs expression in POP tissues was measured by qRT-PCR. Total RNA were extracted from 24 ligament specimens by TRIzol reagent (Invitrogen; Thermo Fisher Scientific, Inc.). CDNA was reverse transcribed with M-MLV reverse transcriptase (Invitrogen, Shanghai, China) following the supplier protocol. PCR was conducted with the StepOne (Applied Biosystems, Shanghai, China). Primers that were producted from BioSune Biotechnology Co., Ltd. The quantification of lncRNA were achieved to comparing with the β-actin expression level as the internal control.

### 2.8. lncRNA-mRNA network

The network analysis was essential to dysregulated lncRNAs and their targeted mRNAs based on the STRING database. We did this by taking objective genes from the database. Significant correlation pairs of lncRNA and mRNA were selected to construct the network. The network was visualized by Cytoscape software.

### 2.9. Statistical analysis

Every experiment was replicated at least 3 times. All specimens were described with means ± standard deviation. All statistical calculations were performed with SPSS 19.0. Student *t* test and 1-way ANOVA as statistics method was used to analyze significance levels of 2 groups. Statistical significance was defined as *P <* .05.

## 3. Results

### 3.1. lncRNAs identification

The lncRNA expression profile was detected by high-throughput RNA-seq. Average of 10.5 million raw sequencing data were obtained from both tissues with the Illumina HiSeq platform. Excluding the low-quality and adaptor sequences, there are 1,006,596,942 clean reads accounting for 151.01 Gb to be analyzed.

Subsequently, 5 steps were used to screen the transcripts for identify the confidently expressed lncRNAs. According to the feature of the lncRNA particular structure, the final results yielded 289 novel lncRNAs (Fig. 1A) including 256 (88.6%) lncRNAs and 33 (11.4%) antisense lncRNAs (Fig. 1B). coding-non-coding-index, coding potential calculator, Pfam-scan, and Pfam-sca and phylogenetic codon substitution frequency were used to eliminate transcripts with coding capacity (Fig. 1C).

### 3.2. Differential expression and clustering analyze of lncRNAs and mRNAs

**Figure 2.**
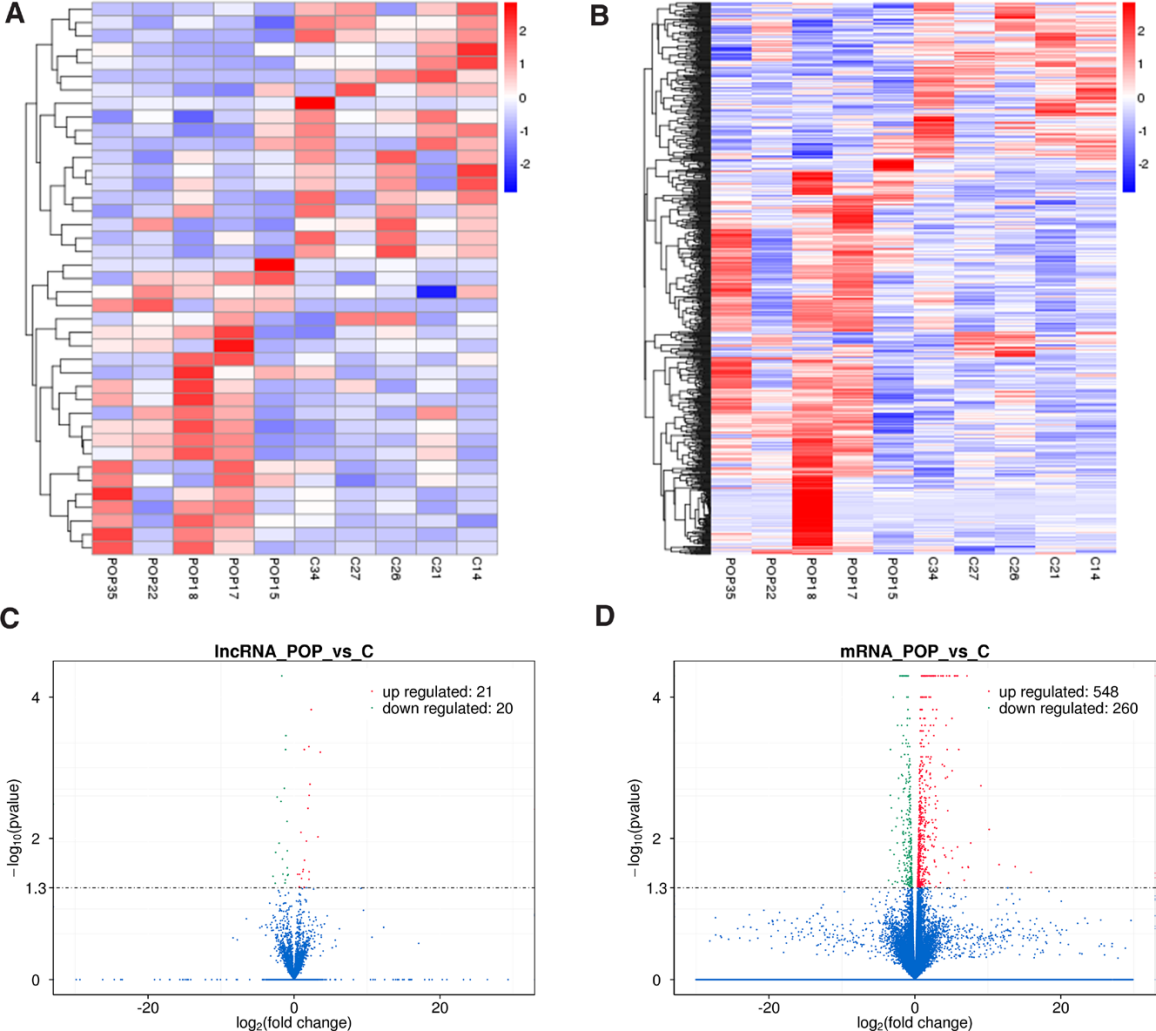
Aberrantly expressed of lncRNAs and mRNAs in POP and non-POP uterosacral ligament tissues. (A) Volcano plot of differentially expressed lncRNAs between POP and control group. (B) Volcano plot of differentially expressed mRNAs between POP and control group. (C) Hierarchical cluster heat map of the expression profiles of differentially expressed lncRNAs. (D) Hierarchical cluster heat map of the expression profiles of differentially expressed mRNAs. Up-regulated lncRNAs and mRNAs are denoted in red and down-regulated in blue (*P ≤* .05 and fold change ≥ 2.0). lncRNA = long noncoding RNA, POP = pelvic organ prolapse.

Expression profiles of lncRNAs and mRNAs in patients with POP and non-POP groups were terribly different. In patients with POP, add up to 41 lncRNAs (21 up-regulated and 20 down-regulated) and 808 mRNAs (548 up-regulated and 260 down-regulated) exhibited peculiarly expression versus the controls (Fig. 2A and B). Hierarchical cluster analysis of differential expression mRNAs and lncRNAs was performed. Heat maps indicated clearly self-segregated clusters between patients with POP and controls (Fig. 2C and D). The major ten up-regulated and down-regulated lncRNAs and mRNAs are shown in Tables 1 and 2, respectively.

**Table 1:** Top 10 differentially expressed lncRNAs.

Gene name	log2FC	*P* value
Up-regulated lncRNAs		
LA16c-359F1.1	inf	.00385
FLJ12825	3.61398	.0006
LINC01605	3.28469	.0095
RP11-753A21.1	2.34882	.00015
LINC00968	2.19146	.0017
FENDRR	2.07688	.02975
MIR503HG	2.07344	.00245
CTD-2231H16.1	2.06723	.03795
MIR503HG	2.06496	.0005
MIR646HG	1.91501	.00375
Down-regulated lncRNAs		
LINC01088	−2.87239	.03445
CTD-2033D15.2	−2.57391	.0433
RP11-219A15.4	−2.50683	.01575
RP11-1100L3.8	−2.31463	.0026
LINC01139	−2.03601	.0117
XLOC_060057	−1.79137	.003
LINC00890	−1.66137	5.00E−05
LINC00473	−1.59372	.01965
LINC00473	−1.51974	.03195
CTC-250I14.6	−1.30837	.00195

lncRNA = long noncoding RNA.

**Table 2:** Top 10 differentially expressed genes.

Gene name	log2FC	*P* value
Up-regulated LncRNAs		
TMPRSS11B	inf	5.00E−05
PLA2G4D	inf	5.00E−05
C10orf99	inf	5.00E−05
SERPINB3	inf	.03105
SERPINB3	inf	.0361
TMPRSS11E	inf	5.00E−05
SPINK5	15.9277	.03025
TMEM79	13.7464	.0251
KRT4	11.5436	.02355
KRT13	10.1788	.00745
Down-regulated LncRNAs		
ITGAV	−3.61252	.0394
FCGR3B	−3.40397	.0146
NCMAP	−3.35447	.00245
PIGR	−3.34005	.00055
WIF1	−3.23235	.0037
CRABP1	−3.13899	.00775
NR4A3	−3.08601	.0443
NR4A1	−2.94076	.0001
CHGB	−2.8684	.0169
KIF1A	−2.70038	.041

lncRNA = long noncoding RNA.

### 3.3. GO classification and KEGG pathway of deregulated lncRNAs

**Figure 3.**
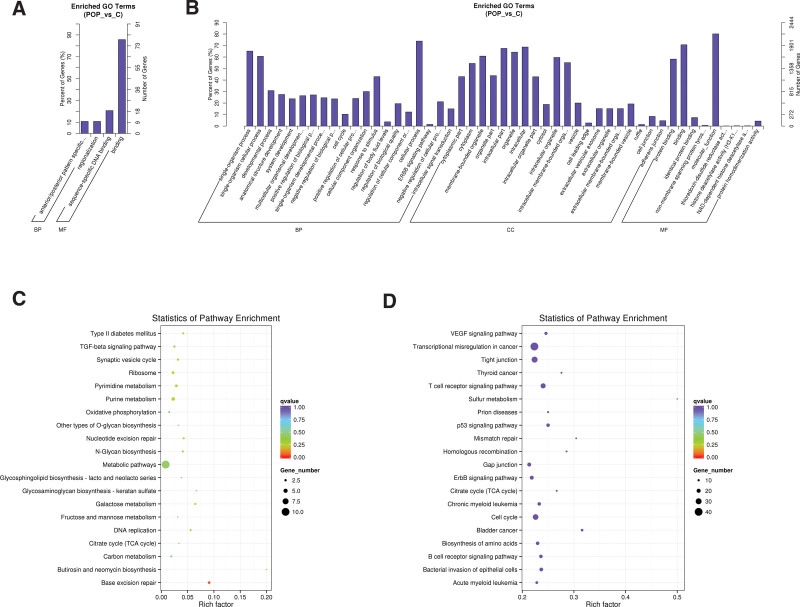
GO and KEGG pathway enrichment for gene functions of predicated lncRNAs in POP patients and controls. (A) GO enrichment analysis of differential expressions of lncRNAs cis-regulation genes. (B) GO enrichment of differential expressions of lncRNAs trans-regulation genes. (C) KEGG pathway analysis of differential expressions of lncRNAs cis-regulation genes. (D) KEGG pathway analysis of differential expressions of lncRNAs trans-regulation genes. GO = gene ontology, lncRNA = long noncoding RNA, KEGG = Kyoto encyclopedia of genes and genomes, POP = pelvic organ prolapse.

To further evaluate the role of differential expression lncRNAs, we performed GO classification and KEGG pathway enrichment to better study the role of differential expression lncRNAs in target genes by cis- and trans-acting. GO enrichment analysis showed that the important roles mainly attended to anterior/posterior pattern specification, regionalization, sequence-specific DNA binding, and binding in lncRNAs cis-acting genes between POP and control group. In regard to lncRNAs trans-acting genes, they were highly enriched in protein binding, single-organism cellular process, cytoplasmic part, cell cycle, and so on. The critical biological terms were shown in Figure 3A and B. KEGG pathway analysis disclosed that the cis-regulation genes of differential expression lncRNAs were mainly referred to Base excision repair, TGF-beta signaling pathway, Metabolic pathways, Cell cycle, p53 signaling pathway, and VEGF signaling pathway were in lncRNAs trans-regulation genes. The remainder part was indicated in Figure 3C and D. These findings suggested these lncRNAs involved in human health and disease regulations, might relate to POP formation.

### 3.4. GO and KEGG analysis of deregulated mRNAs

**Figure 4.**
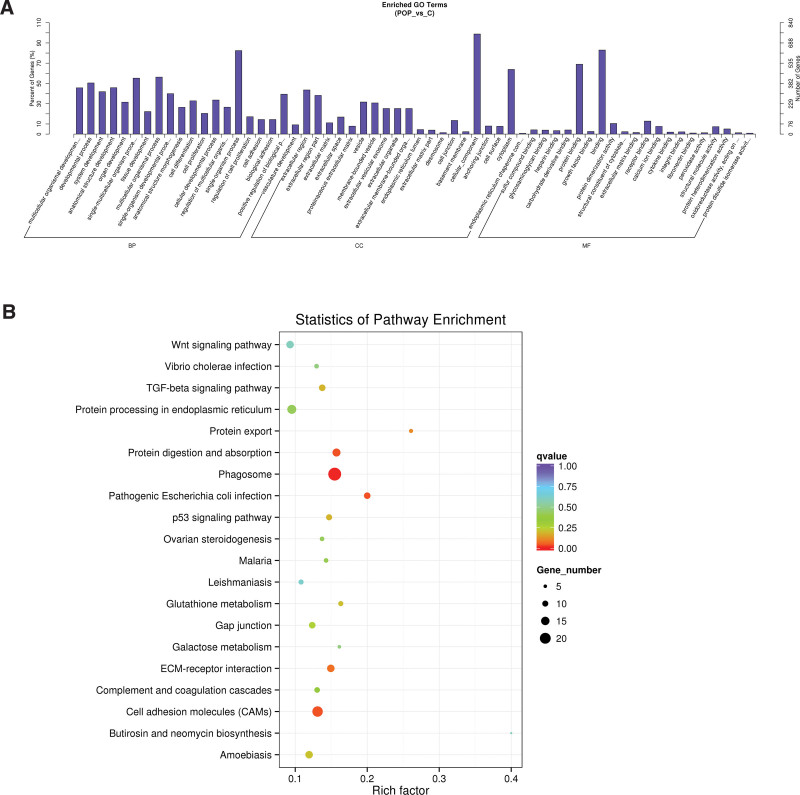
GO enrichment and KEGG pathway analysis of differentially expressed mRNAs in POP patients and controls. (A) Top 20 GO terms enriched among differentially expressed mRNAs. (B) The top 20 pathways enriched among differentially expressed mRNAs. GO = gene ontology, KEGG = Kyoto encyclopedia of genes and genomes, POP = pelvic organ prolapse.

Differential expression mRNAs were conducted to discover their function and connection by GO terms and KEGG enrichment. The significant enrichment processes of GO analysis were presented in Figure 4A. The great majority of these genes were primarily related to the next items: extracellular matrix (ECM), cell differentiation, cell proliferation, and cellular developmental process. At the same time, according to KEGG pathway analysis, ECM-receptor interaction, p53 signaling pathway and cell adhesion molecules were the most significantly enriched pathways (Fig. 4B).

### 3.5. qRT-PCR verification

**Figure 5.**
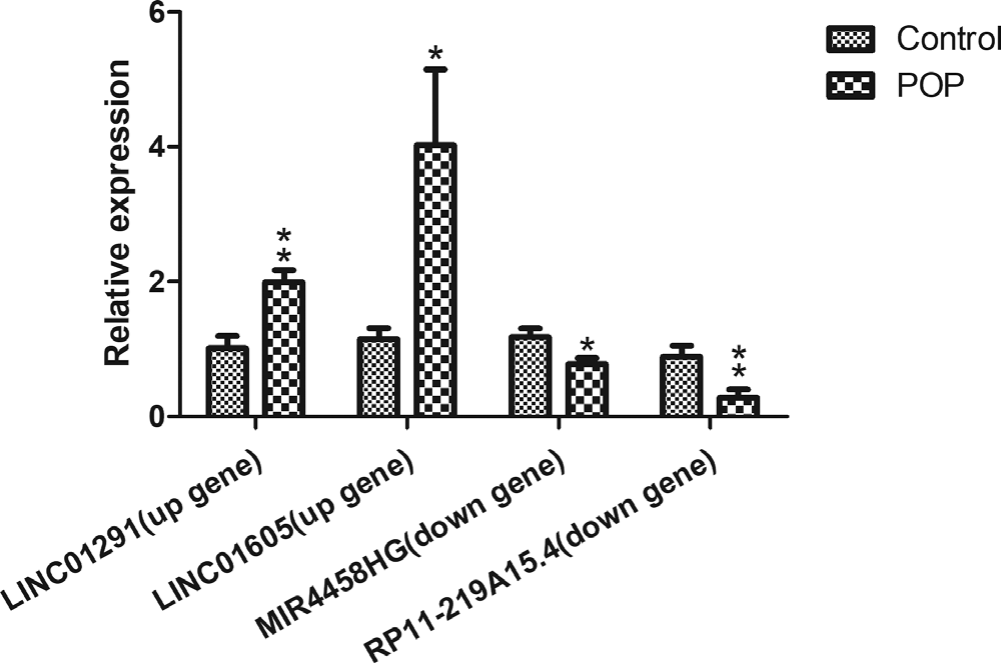
Quantitative RT-PCR validation of the expression of 4 randomly chosen lncRNAs. The expression levels of all chosen transcripts in hUSLs were significantly different compared to the control group. * represents a *P* value of (*P* < .05). ** represents a *P* value of (*P* < .01). hUSL = human uterosacral ligament, lncRNA = long noncoding RNA, RT-PCR = real-time polymerase chain reaction.

Our bioinformatics analysis and RNA-sequencing results were validated with qRT-PCR. Two elevated expression lncRNAs (LINC01291, LINC01605) and 2 reduced expression lncRNAs (MIR4458HG, RP11-219A15.4) in POP specimens were assessed by qRT-PCR. In accordance with the sequencing results, the expression quantity of 4 lncRNAs in the hUSL samples was significantly different between POP and control group (Fig. 5).

### 3.6. The network of lncRNAs and targeted mRNAs

**Figure 6.**
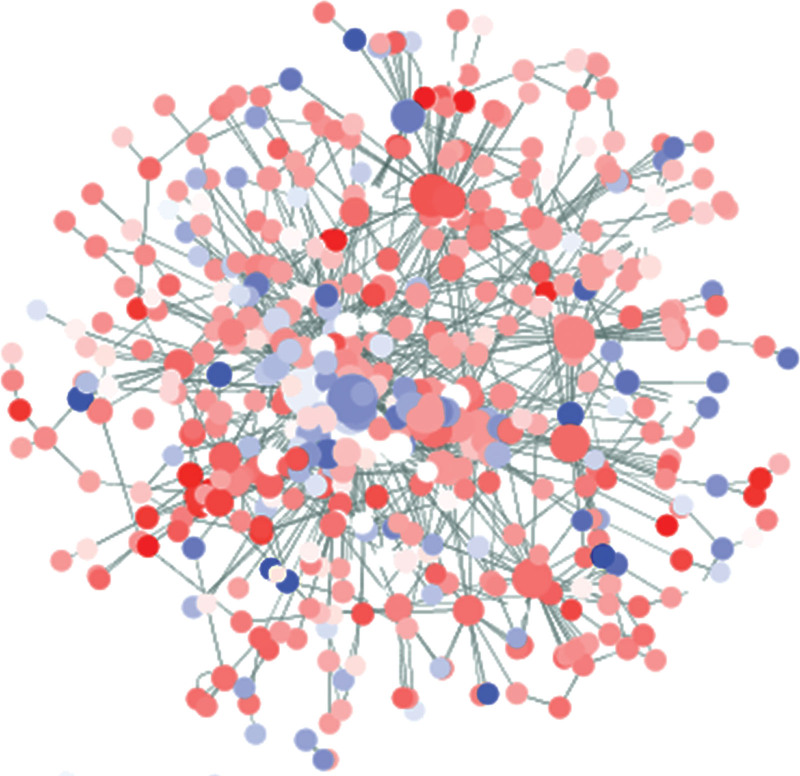
A network consisting of differentially expressed lncRNAs and mRNAs, and the extended network based on lncRNA-mRNA co-expression and co-location. The nodes represent lncRNAs and mRNAs. Node color reflects the expression values (red for higher expression and green for lower expression). lncRNA = long noncoding RNA.

**Figure 7.**
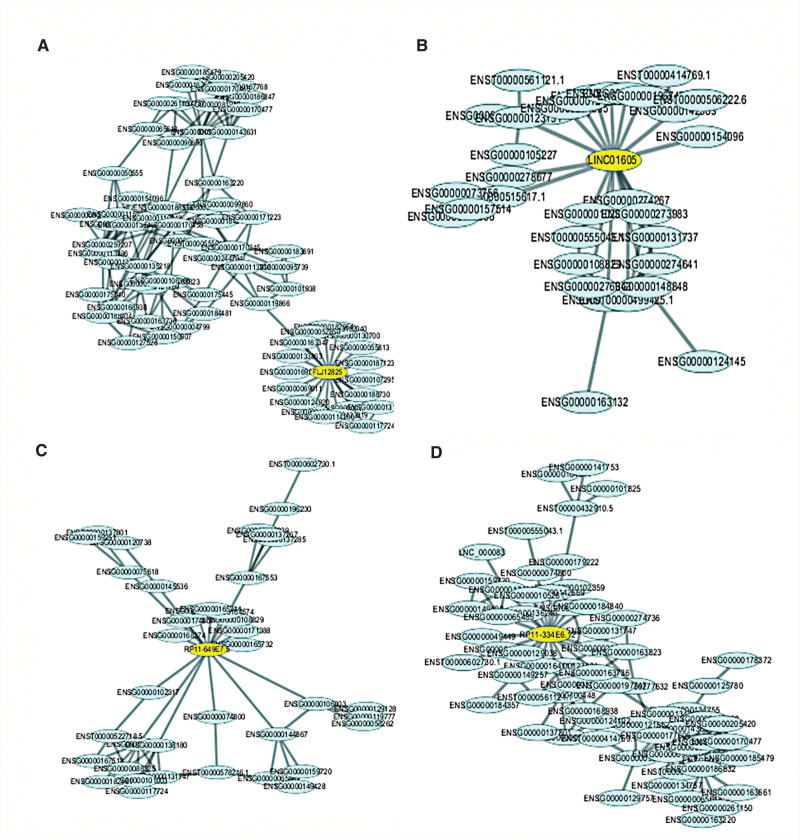
The sub-network of genes connectivity degrees in different module. Four lncRNAs were centered in the 4 modules. (A): FLJ12825. (B): LINC01605. (C):RP11-649E7.5. (D): RP11-334E6.12. lncRNA = long noncoding RNA.

In order to further reveal the communications and associations among lncRNAs and mRNAs in hUSLs, the regulatory co-expression network was built according to the differentially expressed lncRNA and mRNA. The system contains differently expressed lncRNAs and their targeting mRNAs (Fig. 6). Up and down regulated RNAs were indicated by nodes, and the regulatory correlations between lncRNAs and mRNAs were indicated by lines. In the current research, we selected the largest 4 sub-networks for our analysis. Among these, 4 lncRNAs were centered in the 4 modules (Fig. 7A–D). These findings suggest that the important role in the pathogenesis of POP was established for these lncRNAs.

## 4. Discussion

POP constitutes a “hidden epidemic” and its prevalence has gradually been seen in younger women.^[19]^ POP development is a variety of multiple risk factors acting on the pelvic floor and is incompletely understood although recent evidence has suggested that besides environmental factors and structural modifications, genetic predisposition plays a critical role.^[20]^ The molecular mechanisms underlying POP are not very clear at present. Recently, thousands of lncRNAs were identified and described in numerous biological processes, including cell cycle progression, apoptosis, cell invasion and proliferation, and so on.^[21,22]^ An important part of the pelvic support system is the uterosacral ligament.^[23]^ Şükrü et al found the relationship between high-grade POP with serum NGF level correlations and found a positive correlation with the anterior and apical compartment parameters of Pelvic Organ Prolapse Quantitation System, that may occur as a result of a weakening of the ligaments or tissues.^[24]^ Changes in the pelvic connective tissues, including the uterosacral ligaments, may contribute to POP. A recent study of uterosacral ligament biopsies from women with and without POP demonstrated a significant reduction in elastin content in women with POP.^[25]^ In the present experiment, the differential expression profiles of lncRNA and mRNA in the uterosacral ligament of POP were detected using RNA-seq analysis. For all we know, this is the first research checking gene expression profiles by sequencing in uterosacral ligament tissues, describing lncRNAs and mRNAs associated with POP.

A total of 41 lncRNAs (21 up-regulated and 20 down-regulated) were differentially expressed in the POP group matched with the control group, identified by RNA-Seq. The upregulation of LINC01291, LINC01605, and LINC00968 as well as downregulation of MIR4458HG, RP11-219A15.4 were verified to qRT-PCR analysis, which was conforming to the RNA-seq results. Among differentially expressed genes, lncRNA LINC01605 had been detected to increase in POP tissues and promoted the proliferation, migration, and invasion of bladder cancer cells via activating EMT signaling pathway and up-regulating MMP9 expression.^[26]^ More importantly, MMP9 protein expression was increased in stretch-conditioned media from POP women compared to control women.^[27]^ LncRNA LINC00473, a highly conserved lncRNA that has been reported as one of the down-regulated lncRNAs in various types of cancer, including cervical cancer^[28]^ and gastric cancer,^[29]^ and plays a role in cell migration and invasion.

Studies in lncRNA of POP are still at their infant stage, and we still do not know all the functional annotations of each lncRNA and their combinatorial requirements. The ways of lncRNAs functional projection are mainly through the study of their correlated biological pathways and related protein coding genes.^[30]^ Therefore, we further explored that 808 mRNAs were differentially expressed in tissues of POP patients, containing 548 up-regulated and 260 down-regulated. These mRNAs were divided into 2 categories according to the cis-regulation or trans-regulation effect and subsequently conducted on GO and KEGG pathway enrichment analyses.^[31]^

In this research, GO enrichment analysis indicated that significantly differentially expressed mRNAs were enriched in cellular components like ECM and biological process like cell proliferation and differentiation. lncRNA targeted co-located mRNAs were associated with sequence-specific DNA binding, regionalization, and binding, and lncRNA targeted co-expressed mRNAs were associated with cell cycle and cell death. The degradation and remodeling of ECM components serve a major role in POP.^[32,33]^ ECM is a complex macromolecular structure that has a crucial influence on tissue and organ morphogenesis. Additionally, interactions between cells and the ECM control a plethora of cell behavior such as adhesion, differentiation, proliferation, and apoptosis, which are completely participated in tissue remodeling.^[34,35]^ It suggested that the reconstruction of ECM induced by lncRNAs may regulate the pathogenesis of POP.

KEGG pathway analysis showed up or down regulated mRNAs were mainly enriched in pathways such as ECM-receptor interaction, Wnt signaling pathway, and p53 signaling pathway. KEGG pathway analysis found that those lncRNAs were mainly associated with the TGF-beta signaling pathway, tight junction, metabolic pathways, and VEGF signaling pathway, which were partially in agreement with previously published studies in the research of POP. Canonical Wnt16 signaling transduction pathway has been found to be inhibited in the fibroblasts of POP patients resulting in lower growth and proliferation activity and reduced secretion of collagen.^[36]^ There also existed that TGF-β1 expression positively correlated with MMP-9 expression and the TGF-β1 expression, but not MMP-9 expression, also significantly increased with age.^[37]^ Research has shown that hypoxia may require increased apoptosis of cardiomyocytes via the activated p53 signaling pathway.^[38]^ The previous study of our research group showed that expression levels of HIF-1α and apoptosis cells were significantly increased in the POP group.^[39]^ Therefore, HIF-1α may promote fibroblast apoptosis by activating the p53 signaling pathway, and then reducing the production of collagen.

On the other hand, there are also some limitations to this research. Firstly, there was a small sample size of USL tissues in RNA-seq. Secondly, our results have been tested only in small scale data sets. Further validation studies of the lncRNA in other larger populations are also needed. Besides that, this study did not excavate specific functions of these differentially expressed mRNAs and lncRNAs in POP. There will be more focus in the years to come on the functions of those genes. Finally, although there is a difference in lncRNA expression between POP specimens and controls, it is unclear whether this difference is a cause or a consequence of POP. It is not difficult to assume that the occurrence of POP causes various stresses to tissues of the pelvic (e.g., USLs), and this stress can alter the expression of various RNAs. Follow-up relevant experimental evidence is needed.

In summary, we first analyzed the expression of lncRNA and mRNA expression in uterosacral ligament tissue of POP using the RNA-seq approach. GO and KEGG enrichment analyses allowed us to further analyze the potential functions of differentially expressed mRNAs regulated by the lncRNA. Furthermore, the lncRNA-mRNA network in the pathogenesis of POP was constructed and analyzed. The pathophysiological relationship between lncRNAs and POP needs to be studied further. lncRNAs and their target genes were predicted to help in understanding the regulatory roles of lncRNAs in POP development, and the lncRNAs associated with the HIF-1α were revealed. We will further study the underlying mechanisms in POP through increasing clinical samples.

## Author contributions

**Conceptualization:** Ping Li.

Data curation: Ping Li.

Formal analysis: Ping Li.

Funding acquisition: Peishu Liu.

Investigation: Xinrui Zhao, Lu Wang.

Methodology: Xinrui Zhao, Lu Wang.

Project administration: Peishu Liu, Lu Wang.

Resources: Peishu Liu, Ping Zhang.

Supervision: Ping Zhang.Validation: Xinrui Zhao.

Writing – original draft: Xinrui Zhao.

Writing – review & editing: Ping Zhang.
